# ABCG2 confers promotion in gastric cancer through modulating downstream CRKL in vitro combining with biostatistics mining

**DOI:** 10.18632/oncotarget.14128

**Published:** 2016-12-23

**Authors:** Junqing Wang, Zhou Yunyun, Lu Wang, Xuehua Chen, Zhenggang Zhu

**Affiliations:** ^1^ Department of Surgery, Ruijin Hospital, Shanghai Jiao Tong University School of Medicine, Shanghai 20025, People's Republic of China; ^2^ Shanghai Key Laboratory of Gastric Neoplasms, Ruijin Hospital, Shanghai Jiao Tong University School of Medicine, Shanghai 20025, People's Republic of China; ^3^ Shanghai Institute of Digestive Surgery, Ruijin Hospital, Shanghai Jiao Tong University School of Medicine, Shanghai 20025, People's Republic of China; ^4^ Department of Data Science, University of Mississippi Medical Center, Jackson, MS 39216, USA; ^5^ McArdle Laboratory for Cancer Research, University of Wisconsin School of Medicine and Public Health, Madison, WI, 53706, USA

**Keywords:** ABCG2, CRKL, cell proliferation, cell apoptosis, gastric cancer

## Abstract

ABCG2, member of ATP-binding cassette (ABC) transporter family, is known as crucial regulator related to multi-drug resistance in human tumors and has recently been putatively studied as human carcinoma cell biomarker. While, effects of ABCG2 on human gastric cancer (GC) has not been illustrated thoroughly. In this study, by applying biostatistics mining methods, we observed that ABCG2 is frequently aberrantly expressed in GC patients through exploring dataset of GSE19826 in NCBI GEO database. Contemporary, extreme up-regulation of ABCG2 was discovered in both GC specimens and cell lines of our center, from which we observed high level of ABCG2 associated with GC clinicopathologic features and poor outcomes. Depletion of ABCG2 in MKN-45 GC cells, the cell proliferation was significantly impacted along with cell cycle arrest, and cell apoptosis was induced. Interestingly, combined with data mining of NCBI database, CRKL, a pivotal GC promoter, presents a significant positive correlation with ABCG2. And the expression of CRKL in GC cells was obviously affected through ABCG2 depletion. Simultaneously, over-expression of CRKL in MKN-45 cells significantly rescued most of the phenotypes induced by ABCG2 depletion. Thus, we suggest that ABCG2 is a potential biomarker and target upstream CRKL, which could be further studied for GC diagnosis and therapeutic treatment

## INTRODUCTION

Gastric cancer (GC) is one of the most common human carcinomas around the world, contributing to 10% newly diagnosed cases per year with relatively high mortality in patients of advanced stages [1]. The challenges of finding associated gastric tumor markers and understanding the mechanisms of GC initiation, progression and metastasis, are keys to detecting GC in early stages and target treatment [2, 3].

ATP-binding cassette subfamily G, member 2 (ABCG2), is a member of the ATP-binding cassette (ABC) transporter family. It was firstly discovered in a process involving in multidrug resistance (MDR) from doxorubicin-resistant human MCF-7 breast cancer cells [4–6], and was also reported to induce sorafenib resistance in hepatocellular carcinoma (HCC) cells [7]. Further investigation indicated that ABCG2 is over-expressed in various tumor cells [8, 9]. Recently, ABCG2 has been studied as a potential marker of cancer stem cells (CSCs) in diverse human malignances, associated with tumor initiation, maintenance, relapse, metastasis and MDR, which suggests ABCG2 a hopeful target in cancer treatment [9–13]. As reported by Jiang, et.al, ABCG2 might present as a highly expressed stem cell biomarker in GC cells, which is associated with poor- or un-differentiated cell status [14]. However, the evidence that ABCG2 affects GC cells throughout the tumorigenic process is not adequate thoroughly in GC for translational application for clinic.

In this study, we firstly mined and discovered aberrant expression of ABCG2 in GC patients from NCBI database, which directed us to conduct further investigation in both GC tumor tissues and cell lines. As observed, ABCG2 is over-expressed in GC tumors specimens and cell lines. The highly expressed ABCG2 is associated with poor outcomes of GC patients with more advanced clinicopathologic features. By depleting ABCG2 in one of the GC cell lines, MKN-45, the cell growth and apoptosis status was significantly affected. And interestingly, CRKL, a crucial promoter of GC, was subsequently down-regulated in MKN-45 cells. Thus we wondered if there exists regulation between ABCG2 and CRKL. As what we found, ABCG2 is a potential regulator of downstream CRKL, and ectopic introduction of CRKL into MKN-45 cells significantly rescues the phenotypes induced by ABCG2 depletion. Thus, what we discovered here indicates ABCG2 as a promoter in GC cells through regulating CRKL.

## RESULTS

### ABCG2 is up-regulated through NCBI GEO database mining and highly expressed in GC tissues

**Figure 1:**
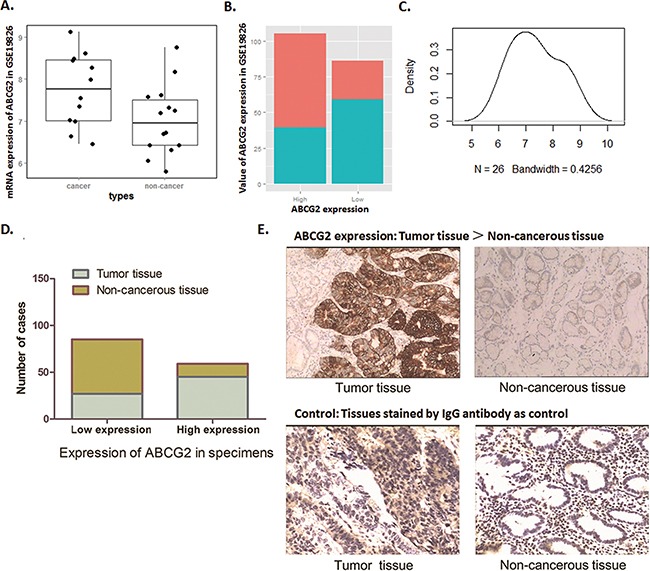
Biostatistics analysis of NCBI database and IHC examination show highly ABCG2 expression in GC patients **A~C**. Dataset GSE19826 was downloaded in NCBI GEO database and analyzed. (A) Box Plot of dataset GSE19826, which demonstrates that ABCG2 expressed a significantly high mRNA level in GC cancer tissues than that of in non-cancerous tissue (*P* < 0.05). (B) Value of total ABCG2 expression of dataset GSE19826. In high ABCG2 expression group, tumor samples contribute the dominant portion, and in low ABCG2 expression group, non-cancerous tissues contribute to the dominant portion. (C) Density plot of the distribution of ABCG2 expression in dataset GSE19826. **D**. Immunohistochemical analysis showed that expression of ABCG2 in GC tumor tissues was over-expressed in most of the tumor tissues (43/72), while, was expressed at significantly lower level in most of the adjacent non-cancerous tissues (58/72). The expression of ABCG2 in tumor tissues was more frequently and significantly higher than that of the adjacent non-cancerous tissues (*P* < 0.01). **E**. Representative graph immunohistochemistry analysis (400×). Specimens stained without primary antibody was used for control. The expression of ABCG2 in GC tumor specimens was significantly higher that of the adjacent non-cancerous tissues.

The analysis result from Oncomine for GSE19826 was shown in Figure 1A, ABCG2 mRNA level in GC tumor tissues is significantly higher than the paired adjacent normal gastric mucosa, which indicate a frequent over-expression of ABCG2 in GC tissues. Divided as high ABCG2 expression group and low ABCG2 expression group, tumor samples contribute to the dominant portion of high ABCG2 expression group, while in low ABCG2 expression group, non-cancerous tissues contribute to the dominant portion (Figure 1B). The density blot demonstrates the distribution of ABCG2 expression in dataset GSE19826 (Figure 1C).

Along with this observation of data mining, we found through IHC detection that ABCG2 presents an obvious higher expression level in tumor tissues of the 72 GC patients compared with the non-cancerous adjacent tissues. Samples of the 72 patients were grouped as ABCG2 ‘high expression’ and ‘low expression’ according to the staining density of IHC. As Figure 1D-1E shown, 62.5% (45/72) of the tumor specimens presented aberrantly high ABCG2 expression, and the cases of ‘low expression’ were counted only 27. For adjacent non-cancerous tis sues, cases of ‘high expression’ were counted only 14 in a relatively lower proportion, which consisted of 19% of the 72 cases, and the cases of ‘low expression’ consisted of almost 81% (58/72). Thus, ABCG2 was highly expressed in tumor tissues compared with the adjacent non-cancerous tissues (*P* < 0.01).

### GC cell lines present high expression of ABCG2 compared with GES-1 cells

**Figure 2:**
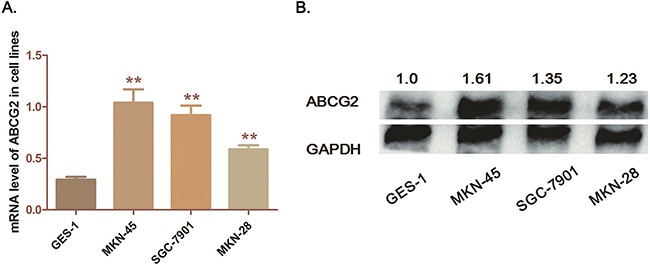
Expression of ABCG2 in GC cell lines **A**. Analysis of transcription level of ABCG2 in cell lines by qRT-PCR. The mRNA levels of ABCG2 in three cell lines (MKN-45, SGC-7901 and MKN-28) was significantly higher than that in GES-1 cells (**P < 0.01, *P < 0.05) **B**. Detection of protein expression of ABCG2 in cell lines by western-blot analysis. ABCG2 was significantly over-expressed in GC cells compared with GES-1 cells. The numbers above the blot indicate normalized protein amounts relative to the negative control, as determined by densitometry. As histograms above shown, MKN-45 cells presented a highest ABCG2 expression among the GC cell lines.

The immortalized gastric epithelium cell line (GES-1) is regarded as control for comparison with the GC cell lines. Three GC cells lines (MKN-45, SGC-7901 and MKN-28) were detected. As qRT-PCR and Western blot analysis demonstrated, both mRNA and protein levels of ABCG2 are significantly higher in the 3 GC cell lines than in GES-1 cells (*P*<0.05) (Figure 2). Among these 3 GC cell lines, MKN-45 cell, which is the poorly differentiated one, presented the highest level of ABCG2. Herein, the high expression characteristic of ABCG2 in GC cells is consistent with the observation through IHC of tumor tissues. And we further selected MKN-45 for ABCG2 depletion.

### Expression characteristic of ABCG2 is correlated with the clinicopathologic features and ABCG2 is an independent prognostic factor of GC

According to the expression status of ABCG2 in GC, we speculated a correlationship between expression level of ABCG2 and the GC clinicopathologic features. As Table 1 shown, we listed the expression characteristic of ABCG2 in 72 GC cases. Among the 72 GC cases, higher expression of ABCG2 presented significant inclination towards larger tumor size (*P* < 0.05), deeper local invasion (*P* < 0.05), more lymph node metastasis (*P* < 0.05) and advanced TNM stage (*P* < 0.05), while it had no significant correlation between ABCG2 and patients’ age, gender, tumor location. These results strongly suggested a significant correlation between ABCG2 expression and the GC clinicopathologic parameters concerning with poor prognosis.

**Table 1:** The correlation between expression characteristic of CRKL and ABCG2 in GC specimens and GC clinicopathologic features

Clinicopathologic parameters	CRKL expression (n=e52)		P *	ABCG2 expression (n=72)		P *
	Low (n=15)	High(n=37)		Low (n=27)	High(n=45)	
**Age (years)**						
≤60	5 (33%)	16 (43%)	0.509	14 (51%)	21 (47%)	0.808
>60	10 (67%)	21 (57%)		13 (49%)	24 (53%)	
**Gender**						
Male	12 (80%)	25 (68%)	0.37	15 (56%)	28 (62%)	0.625
Female	3 (20%)	12 (32%)		12 (44%)	17 (38%)	
**Diameter (cm)**						
≤5	12 (80%)	18 (49%)	0.038	19 (70%)	18 (40%)	0.016
>5	3 (20%)	19 (51%)		8 (30%)	27 (60%)	
**Location**						
Distal third	10 (67%)	23 (62%)	0.76	14 (52%)	31 (69%)	0.209
Middle or proximal third	5 (33%)	14 (38%)		13 (48%)	14 (31%)	
**Histologic Classification**						
Poorly differerntial	7 (47%)	22 (59%)	0.792	11 (41%)	23 (51%)	0.501
Middle/well differerntial	4 (25%)	6 (16%)		9 (37%)	8 (18%)	
Signer ring cell cancer	3 (20%)	6 (16%)		3 (11%)	7 (16%)	
Mucinous asenocainoma	1 (8%)	3 (9%)		4 (11%)	7 (14%)	
**Location invasion**						
T1, T2	6 (40%)	2 (5%)	0.002	15 (56%)	13 (29%)	0.045
T3, T4	9 (60%)	35 (95%)		12 (44%)	32 (71%)	
**Lymph node metastasis**						
No	7 (47%)	4 (11%)	0.004	14 (52%)	10 (22%)	0.019
Yes	8 (53%)	33 (89%)		13 (48%)	35 (78%)	
**TNM stage**						
I, II	7 (47%)	2 (5%)	0.001	15 (56%)	12 (27%)	0.023
III, IV	8 (53%)	35 (95%)		12 (44%)	33 (73%)	

CRKL and ABCG2 expression level associated with clinicopathologic features in GC patients, including age, gender, tumor size, tumor location, histologic classification, local invasion, lymph node metastasis and TNM stage. Statistically significance was assessed by Fisher's exact text.

### Expression of ABCG2 and CRKL is positively correlated in GC

Through analysis of microarray data in GSE19826, we also found that CRKL, which has been verified as a highly expressed gene independently related with poor GC prognosis [15], was presented a significant positive correlation with ABCG2.

**Figure 3:**
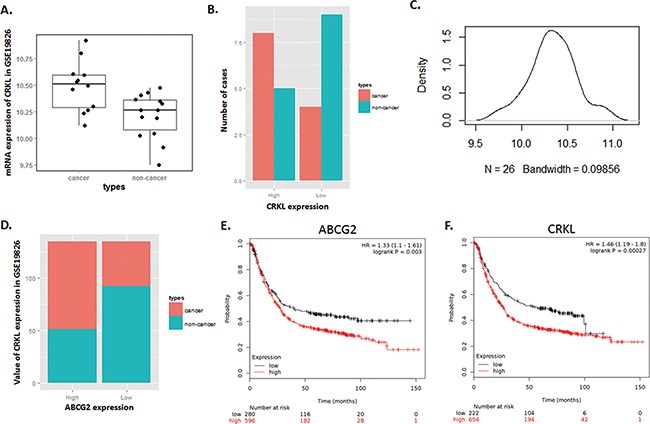
Expression of CRKL in Dataset GSE19826 is positively correlated with ABCG2, and illustrates a high risk of mortality for GC patients with high expression of ABCG2 and CRKL **A~C**. Dataset GSE19826 was downloaded in NCBI GEO database. (A) Box Plot of dataset GSE19826, which demonstrates that CRKL expressed a significantly high mRNA level in GC cancer tissues than that of in non-cancerous tissue (*P* < 0.05). (B) Expression of CRKL in GC tumor tissues was over-expressed in most of the tumor tissues, and was expressed at significantly lower level in most of the adjacent non-cancerous tissue. (C) Density plot of the distribution of CRKL expression in dataset GSE19826. Most of the tumor samples expressed a relatively high level of ABCG2. **D**. Value of total CRKL expression of dataset GSE19826. In high CRKL expression group, tumor samples contribute the dominant portion, and in low CRKL expression group, non-cancerous tissues contribute to the dominant portion. **E**. Patient with highly expressed either ABCG2 or CRKL has a relatively higher risk of mortality (*P* < 0.01).

As shown in Figure 3A-3D, along with the highly expression of ABCG2, CRKL was significantly over expressed in patients included in GSE19826 dataset, which is consistent with our observation in the paired specimens. In Table 1, we listed the expression characteristic of CRKL in 52 GC cases of our previous research to compare with ABCG2 in 72 GC cases of this study. Obviously, either high expression of CRKL or ABCG2 shares a similar correlationship with GC clinicopathologic features indicating poor prognosis.

Survival analysis of ABCG2 and CRKL conducted by online Kaplan–Meier plotter tools was shown in Figure 3E-3F. Patients with high expression of either ABCG2 or CRKL in tumor tissue presented a significant tendency towards poor prognosis and high mortality (*P* < 0.05). These results illustrated that both ABCG2 and CRKL are associated with poor prognosis of GC, and are positively correlated.

### Depletion of ABCG2 suppresses MKN-45 cell proliferation, arrests the cell cycle and induces cell apoptosis

**Figure 4:**
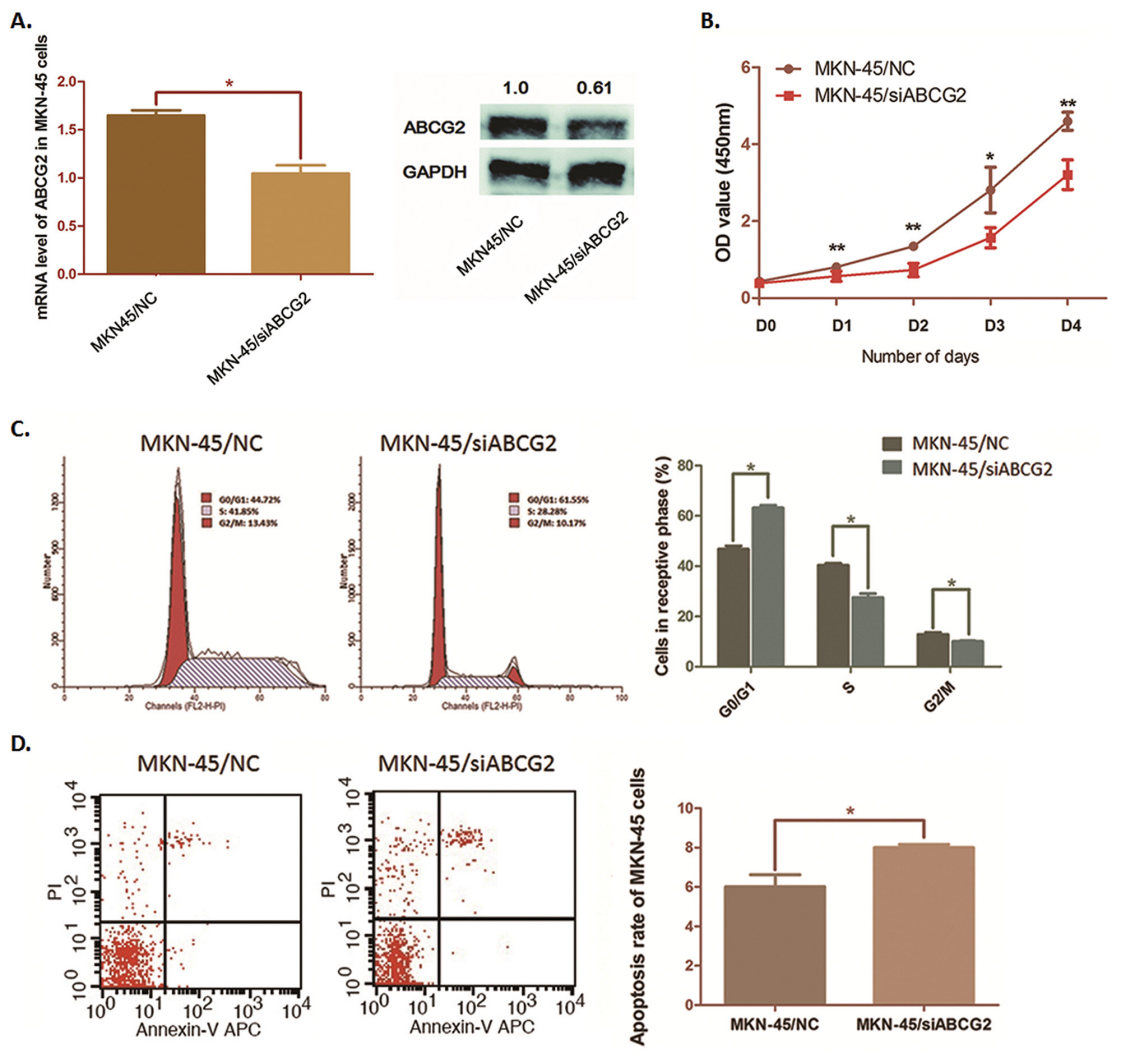
Effects on cell cycle, cell proliferation and cell apoptosis by depleting ABCG2 in MKN-45 cells **A**. MKN45 cells were transfected with pGU6/Neo/siCRKL vector. QRT-PCR assay and Western blot anaylsis were carried out and presented a significant suppression of ABCG2 expression after transfection (*P < 0.05). **B**. The cell proliferation was determined by WST assay when ABCG2 was knocked down in MKN-45 cells. The ability of cell proliferation of MKN-45 cells was significantly decreased by decreasing ABCG2. The results are means of three independent experiments ± SD. (**P < 0.01, *P < 0.05). **C**. Flow cytometry was used to analyze the effect of ABCG2 on cell cycle. Representative histograms describing cell cycle profiles of MKN-45 cells transfected with pGPU6/Neo/shRNA-ABCG2 were presented. The cell cycle of MKN-45 cells was significantly arrested in G0/G1 phase by suppressing ABCG2 as the histogram shown. The results are means of three independent experiments±SD. (*P < 0.05). **D**. Flow cytometry was used to analyze the effect of ABCG2 on cell apoptosis. The apoptosis rate of MKN-45 cells was increased when ABCG2 suppressed. The results are means of three independent experiments±SD. (*P < 0.05).

In this study, MKN-45 cells presented highest ABCG2 expression among the three GC cell lines. By transfecting MKN-45 cells with pGU6/Neo/siABCG2 vectors, we successfully impair the expression of ABCG2 in MKN-45 cells at both mRNA and protein stages (Figure 4A). As the curve of cell proliferation shown in Figure 4B, when ABCG2 depleted, cell proliferation of MKN-45 cells was dramatically suppressed. Flow cytometry analysis illustrated that the cell cycle of MKN-45 cells was significantly arrested at G0/G1 phase along with ABCG2 depression. The percentage of MKN-45 cells in G0/G1 phase was raised from 46.76% to 63.15% (*P*=0.0164), and S phase and G2/M phase were decreased respectively from 40.39% to 27.47% (*P*=0.0310) and from 12.85% to 10.05% (*P*=0.0436) (Figure 4C).

We also observed a significant increase of apoptosis rate in MKN-45 cells from 6.02% to 8.01% (*P*=0.0353) induced by ABCG2 through flow cytometry analysis (Figure 4D). Results above suggested ABCG2 as a promoter in GC cell proliferation and an inducer of cell apoptosis resistance.

### Depletion of ABCG2 down-regulates expression of CRKL

**Figure 5:**
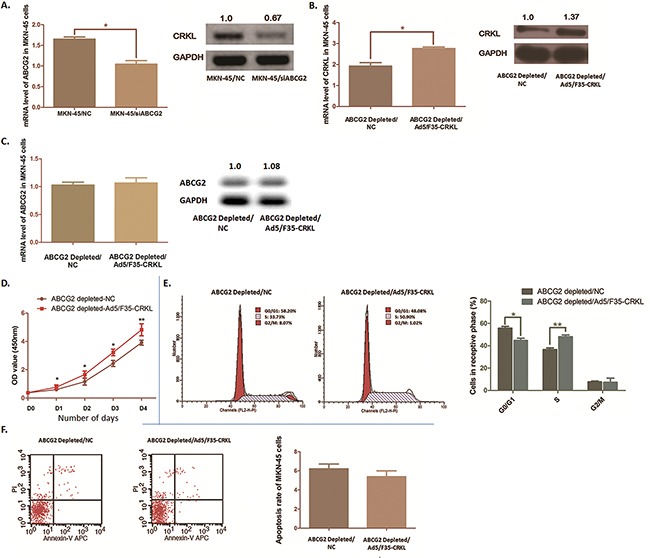
CRKL was modulated by ABCG2 depletion and partly reversed the biological phenomena induced by ABCG2 depletion in MKN-45 cells **A**. QRT-PCR and Western blot anaylsis was carried out. Expression of CRKL at both mRNA and protein stage was suppressed significantly after ABCG2 depletion in MKN-45 cells (*P < 0.05). **B**. Ectopic expression of CRKL was conducted by transfecting recombinant adenovirus Ad5/F35. Significant increase of CRKL mRNA and protein was observed in MKN-45 cells with ABCG2 depletion (*P < 0.05). **C**. Ectopic expression of CRKL in MKN-45 cells with ABCG2 depletion modulated the expression of ABCG2 significantly neigther at mRNA stage nor at protein stage. **D**. WST assay was carried out to determine the cell proliferation ability after CRKL ectopic expression in MKN-45 cells. Cell proliferation suppression induced by ABCG2 depletion in MKN-45 cells was significantly reversed by introducing CRKL. The results are means of three independent experiments ± SD. (**P < 0.01, *P < 0.05). **E**. Proportion of cells in various phases of the cell cycle was detected by Flow cytometry. Ectopic expression of CRKL rescued the cell arrest at G0/G1 phase induced by ABCG2 depletion (*P < 0.05), and the proportion of cells at S phases was obviously increased (*P < 0.05). There observed no significant moderation at G2/M phase (*P*=0.917). The results are means of three independent experiments ± SD. **F**. Flow cytometry was used to analyze the effect of CRKL on cell apoptosis under the depletion of ABCG2. The results are means of three independent experiments ± SD. The apoptosis rate of MKN-45 cells with ABCG2 depletion seems no significant difference when CRKL was ectopically expressed according to the P value (*P*=0.379).

The correlation between ABCG2 and CRKL suggests us a potential regulating relationship between these two genes. To validate this hypothesis, qRT-PCR and Western blot analysis were conducted in ABCG2 depleted MKN-45 cells. As the results demonstrated in Figure 5A, when ABCG2 was down-regulated in GC cells, A dramatic declined of CRKL expression at both mRNA level and protein level was observed. This means ABCG2 is an upstream regulator of CRKL in GC.

### Ectopic expression of CRKL rescues the phenotype induced by ABCG2 depletion

To verify if there exists functional relationship between ABCG2 and CRKL, we introduced CRKL into MKN-45 cells by using recombinant adenovirus Ad5/F35, and validated a significant increase of CRKL expression in MKN-45 cells by using qRT-PCR assay and Western blot analysis (Figure 5B).

As Figure 5C shown, the inhibitory effect on MKN-45 cell proliferation induced by ABCG2 depletion was significantly reversed. Simultaneously, flow cytometry analysis presented a significant decline of the percentage of MKN-45 cells in G0/G1phase from 55.76% to 43.03% (*P*=0.043). Cells at S phase were obviously increased from 36.62% to 48.08% (*P* < 0.01) (Figure 5D). While, no significant evidence was observed that the percentage of G2/M phase was moderated (*P*=0.917). Despite the cell proliferation and cell cycle being affected, the apoptosis rate was not apparently declined when CRKL was over-expressed (*P* =0.379) (Figure 5E). Thus, we illustrated that bio-function of ABCG2 on promoting GC process was partly rescued by over-express CRKL, which indicates ABCG2 is an upstream regulator modulating CRKL in GC.

## DISCUSSION

ATP-binding cassette (ABC) transporter family is a series of proteins exist in multiple cellular membranes from plasma membranes to intracellular components, including endosome, Golgi, endoplasmic reticulum and mitochondria, et.al [16]. ABC transporters apply intracellular ATP hydrolysis, mediate drug efflux and protect cells from the damages induced by xenobiotics and toxins [17, 18]. Characteristics of this contributes a sort of critical mechanism of MDR in human malignancy, and members of this family are frequently over-expressed in cancer [19, 20].

ABC transporters are classified into eight groups from A to H, and two nucleotide-binding domains and two six-pass trans-membrane domains are required for functional structure [21, 22]. In human beings, ABCA, ABCB, ABCD and ABCG classes have been reported carrying functional domains, or existing as half-transporters which play full transporter function in homo- or hetero-dimers formation [23]. Among these genes, ABCG2 is expressed in a wide range of tissues, including brain, placental syncytiotrophoblast, small intestine, and colon, which has been hypothesized as a strong marker participating in malignancy process [24–27]. However, mechanism of ABCG2's biological and molecular functions in GC leaves much unclear.

In this study, first of all, we explored the database of NCBI and aberrant expression of ABCG2 in GC patients was discovered. According to this, we presumed ABCG2 as a specific functional molecule modulated in GC, and then further investigated the expression characteristic of ABCG2 in both GC specimens and cell lines. Immunohistopathological examination revealed a significant higher expression of ABCG2 in 63% (45/72) GC tumor specimens than the adjacent non-cancerous tissues. Likewise, significantly higher expression of ABCG2 was detected in all of the three GC cell lines compared with GES-1 cells. These results validated that ABCG2 is aberrantly up-regulated in GC.

Then, we compared ABCG2 expression of the GC cases with the clinical pathologic features. The results showed that higher ABCG2 expression presents a significant inclination related to larger tumor size, deeper local invasion and tendency of lymph node metastasis. Moreover, high level of ABCG2 was correlated with advanced TNM stages, correlated with poor prognosis. The survival analysis of ABCG2 generated from the publicly available gastric cancer patients’ database (http://kmplot.com) confirmed that high expression of ABCG2 is associated with poor survival. This suggests ABCG2 as a critical molecule is correlated with GC process and prognosis.

MKN-45 cells, which are the poorest differentiated among the three GC cell lines, presented highest ABCG2 expression in this study. So, we selected MKN-45 cells for exploring the function of ABCG2 in GC. Transfection was conducted to deplete ABCG2 in this cell line. Along with ABCG2 decline in GC cells, the cell proliferation was exactly suppressed with a significant arrest of cell cycle in G0/G1 phase. Meanwhile, the apoptosis rate of ABCG2 was significantly increased, which suggests a crippling of cell apoptosis resistance. These results prompt that ABCG2 participates in the cell generation of GC and prevents GC cell apoptosis. Interference of ABCG2 has functional effect on GC diagnose and treatment.

In this study, we used biostatistics methods to explore the potential correlated molecules downstream ABCG2. In dataset GSE19826 from NCBI GEO, we noticed that CRKL is significantly positive correlated with ABCG2 at expression status. CRKL is a kind of adapter protein of the CRK protein involved in various human diseases [28]. Accumulating evidence has verified CRKL as a promoting factor in multiple cancer progress. CRKL participates in regulating the Erk1/2 and PI3K/Akt pathways and induces promotion of cell invasion and migration in breast cancer cells [29]; over-expression of CRKL significantly increases cell proliferation and invasion in two pancreatic cancer cell lines (Capan-2 and Bxpc-3), which suggests a promoting function of CRKL in pancreatic cancer [30]. In our previous research, CRKL was identified up-regulated in multiple GC cell lines, including MKN-45, SGC-7901 and MKN-2 cells [15]. CRKL promotes GC cell proliferation, arrests the cell cycle and significantly induces cell invasion and migration. However, we have not observed any evidence of CRKL effecting on GC cell apoptosis, which probably suggests other mechanism involves with the GC cell apoptosis modulation.

Here, according to the results from biostatistics analysis, higher expression of either ABCG2 or CRKL presents close relationship with more advanced GC stages and poor prognosis. Both of the two molecules show the poor survival rate respectively of GC patients at higher expression level. Interestingly, CRKL was significantly down-regulated in GC cells after ABCG2 depleting. We hypothesized that there is some regulating event between ABCG2 and CRKL. We transfected recombinant adenovirus Ad5/F35 (Ad5/F35-CRKL) into ABCG2 depleted MKN-45 cells to ectopically express CRKL. Up-regulation of CRKL significantly reversed the cell proliferation depression induced by ABCG2 depletion. The portion of cells arrested in the G0/G1 phase was declined simultaneously. Within this modulation, we found that most of the GC cells were distributing at the S phase, but not the G2/M phase through ectopically up-regulating CRKL. However, the cell apoptosis rate decreased by down-regulating ABCG2 could not be significantly rescued. Therefore, we suppose that CRKL acts as one of the downstream effective molecules moderated by ABCG2, which affects GC cell proliferation and rescues the arrest of the cell cycle at G0/G1 phase. However, we believe that there exist other functional molecules downstream of ABCG2, which involves with GC cell apoptosis.

The results we explored in this study strongly indicate a regulating correlation between ABCG2 and CRKL, and also the influence of ABCG2 on cell proliferation and apoptosis. We validated that ABCG2 was a molecule aberrantly up-regulated in GC tissues and cells. The higher level of ABCG2 expression in GC cells was correlated with advanced stages of GC involved with poor prognosis. ABCG2 was a GC promoter affecting cell proliferation and inducing cell apoptosis resistance. Combining with biostatistics analysis, we found the expression of ABCG2 and CRKL is positively correlated, and CRKL is one of the downstream molecules regulated by ABCG2, which promotes cell growth.However, ABCG2 appears to be a mambrane bound molecule, and there is no evidence indicates ABCG2 as a transcription factor in cellular process. The way ABCG2 regulating CRKL is not clear. And the possible mechanism of ABCG2 affecting GC cell proliferation and apoptosis still needs to be illustrated.

## MATERIALS AND METHODS

### Biostatistics mining of NCBI database

The GC patients mRNA expression microarray data were downloaded from GSE19826 in NCBI GEO. There are 26 samples after removing 1 outlier on the quality control with 12 tumor samples vs. 14 normal gastric mucosa samples in this dataset. These data were preprocessed and analyzed by online database tools Oncomine (http://oncomine.org/). The differential expression comparison of ABCG2 between tumor and non-tumor samples was conducted by student's t-test. The correlation between ABCG2 and other potential genes was also detected.

Association analysis for the ABCG2 mRNA and protein expression and 72 cases’ clinicopathologic features was carried out by SPSS 18.0. P values were calculated by paired t-test and Fisher's exact text. P values < 0.05 were considered statistically significant.

For the survival analysis of ABCG2 and CRKL, we use online tool (http://www.kmplot.com/gastric) to generate Kaplan–Meier curves, which include 876 gastric cancer patients with available clinical data. For the expression of the genes, each percentile of expression between the lower and upper quartiles was computed and the best performing threshold was used as the final cutoff for the Univariate Cox regression analysis. Kaplan–Meier survival plot were downloaded from their website and the hazard ratio with 95% confidence and *P* Value were calculated.

### Cell culture and surgical specimens

The GES-1 cell line and four GC cell lines (MKN-45, SGC-7901 and MKN-28) were purchased from Shanghai Institutes for Biological Sciences, Chinese Academy of Science (Shanghai, China). All cells were cultured in RPMI 1640 supplemented with 10% heat-inactivated fetal bovine serum (FBS), 100 ug/ml streptomycin and 100U/ml Penicillin in a humidified cell incubator at 37°C with an atmosphere of 5% CO_2_.

Seventy two pairs of gastric cancer specimens along with the adjacent non-cancerous tissues were collected from GC patients performed radical gastrectomy without preoperative therapy at the Department of Surgery, Ruijin Hospital, Shanghai Jiao Tong University School of Medicine during 2014-2015. Tissues were made into tissue microarray by Outdo Biotech Company, Shanghai, China. Ethical approval was granted by the research medical ethics committee of Ruijin Hospital, Shanghai Jiao Tong University School of Medicine.

### Immunohistochemistry analysis and Western blot analysis

Antibodies against ABCG2, CRKL and GAPDH (Santa Cruz, USA) were purchased, and horseradish peroxidase-conjugated secondary antibody (Abcam, USA) was prepared. Immunohistochemistry analysis was carried out on the tissue microarray. Antibody against ABCG2 was used following the manufactory instruction (1:50), IgG antibody was stained as control. The tissues were assigned blindly to two professional pathologists for examination.

RIPA buffer containing Protease Inhibitor Cocktail (Pierce, USA) was purchased for lysing cells. Protein concentration was measured by using BCA Protein Assay Kit (Pierce, USA). Proteins extracted were electrophoresed and electrotransfered. Antibody against ABCG2 or CRKL (1:1000) and GAPDH (1:5000) were probed, and horseradish peroxidase-conjugated secondary antibody was prepared for further probe. GAPDH was applied as loading control.

### RNA isolation and qRT-PCR assay

Total RNA was extracted from cell lines by using reagent (Invitrogen, USA) following the manufactory instructions. The first-strand cDNAs were synthesized through High-Capacity cDNA Reverse Transcription Kit (ABI, USA). RT-primers of ABCG2 and CRKL were synthesized by Sangon Biotech Company (Shanghai, China) as follow: 1) ABCG2: 5′-TGTGTTTATGATGGTCTGTTGGT-3′(forward) and 5′-CTGTGCAACAGTGTGATGGC-3′ (reverse); 2) CRKL: 5′-CATTCCCGGGCGGCTCTCTC-3′ (forward) and 5′-CACGCCTTAGCCCGGCAGAC-3′ (reverse). Real-time quantitative polymerase chain reaction (qRT-PCR) was carried out according to TagMan Gene Expression Assays protocol (ABI, USA). The PCR program was set as follows: 95°C for 10 min, followed by 32 cycles of 95°C for 15 s, 60°C for 30 s, 72°C for 45 s.

### Cell transfection

We selected MKN-45 cells for further study according to the results of western blot analysis. MKN-45 cells were transfected with pGU6/Neovectors (GenePhrma, Shanghai, China) containing siRNA against ABCG2 by using Lipofectamine 2000 (Invitrogen, USA), and non-containing ones were used as negative control. Cells were cultured and selected in medium containing 400μg/ml G418 (Santa Cruz, USA), and were cultured and maintained in medium containing 200μg/ml G418.

Recombinant adenovirus Ad5/F35 (Ad5/F35-CRKL) was constructed for ectopically expressing CRKL in MKN-45 cells to investigate the rescue effect on the phenotypes caused by ABCG2 depletion (GenePhrma, Shanghai, China). Ad5/F35-Null was used as negative control. Stable transfected cells above were validated by qRT-PCR and western blot analysis compared with the negative control cells.

### Cell proliferation assay and cell cycle analysis

Transfected cells were collected in an exponential growth phase and were ethanol fixed. 1×10^4^ transfected MKN-45cells, including negative control ones, were respectively cultured in 96-well microtiter plates in triplicate and incubated for 5 days at 37°C with an atmosphere of 5% CO_2_. OD was measured by WST (Water-soluble tetrazolium salt USA) assay by using CCK8 Assay Kit (Dojindo, Japan) according to the protocol. The curves of cell proliferation were plotted.

RNaseA treatment and Propidium Iodide staining were carried out. Cells were detected under flow cytometry by FACSCalibur (Becton Dickinson, USA). Cell populations at the G0/G1, S and G2/M phases were quantified by Modfit software (Becton Dickinson, USA) excluding a calculation of cell debris and fixation artifacts.

### Cell apoptosis analysis

Cell apoptosis rate was calculated by using c Apoptosis Detection Kit I (BD Pharmingen, USA) according to the product instructions. Stable transfected MKN-45 cells were washed by PBS and resuspended in 1×Binging Buffer at a concentration of 1×10^6^ cells/ml. Five microliter of APC and 5μl of PI were added to 100μl of cell suspension and incubated in dark for 15 min. Then 400μ1×Bingding Buffer was added. The apoptosis was analyzed by flow cytometry (Becton Dickinson, USA) and Annexin V-APC-positive and PI-negative cells were considered apoptosis.

### Statistical analysis

Statistical analysis for 72 specimens in Table 1 was carried out by SPSS 18.0 and GraphPad Prism 5.0. *P* values were calculated by paired t-test and Fisher's exact text. Those *P* values < 0.05 were considered statistically significant.
